# Effect of surface treatment on physico-mechanical properties of bagasse fiber reinforced biocomposites and their applications: a critical review

**DOI:** 10.1039/d6ra07233j

**Published:** 2026-09-24

**Authors:** Benudhar Parida, Bijnyan Ranjan Das, Ajaya K. Behera

**Affiliations:** a Department of Chemistry, School of Basic Sciences and Humanities, Odisha University of Technology and Research Bhubaneswar Odisha-751029 India; b Department of Chemistry, Utkal University Bhubaneswar Odisha-751004 India ajayabehera@utkaluniversity.ac.in +91 0674 2581850 +91-9938956715

## Abstract

In response to rising environmental concerns, research into biodegradable composites as long-term alternatives to synthetic plastics has advanced. This review focuses on important problems associated with bagasse fiber surface treatment, thermoplastic and thermoset resin reinforcement, and the usage of bagasse fiber reinforced composites in various sectors. The surface treatment of the fiber improved its compatibility, allowing the production of thermoplastic matrix-based biocomposites that adhere to green chemistry principles. These composites demonstrated promising mechanical properties, with tensile strength ranging from 4–85 MPa and flexural strength in the range of 26.2–210 MPa. They exhibit moderate hydrophobicity, absorbing 10–25% water after 24 hours, and maintain thermal stability up to 250 °C. Bagasse fiber reinforced composites are feasible for modern engineering applications and other industrial uses because of these qualities. This study highlights fiber surface treatment as a workable, low-impact method for waste valorization in addition to addressing current performance and environmental deficiencies. The review concludes by examining various product uses, present issues, and potential directions for the development of environmentally friendly composite technology.

## Abstract 2

Development, characterization, and applications of bagasse fiber reinforced composite.

## Graphical abstract

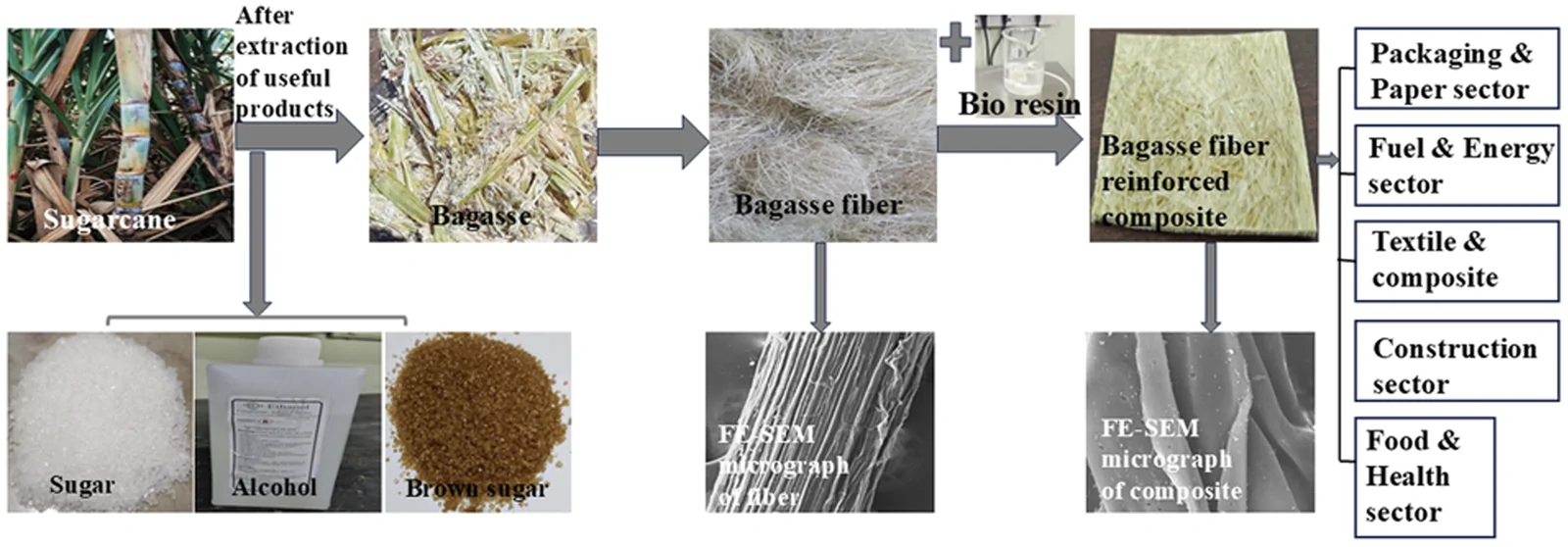

## 1 Introduction

The world is currently dealing with a number of complex problems, such as pollution due to thermoplastic, seasonal instability, floods, earthquakes, sea level rise, and global warming. Researchers, engineers, and industrialists are under pressure to manufacture green materials and new product design employing these materials either fully or partially degradable. The issue of biodegradable waste management and environmental requirements greatly impact scientific research on eco-composite materials, which readily decompose or bio assimilate easily.^1–7^ Scientific research on eco-composite materials is heavily influenced by the issue of biodegradable waste management and standards for a safer and cleaner environment. The most recent advancements in research on green/bio composite materials can also be attributed to the abundance of natural fiber and any other available agro-wastes. Biocomposites are central to sustainable materials development because they combine renewable feedstocks, reduced environmental impact, and functional adaptability. They not only mitigate ecological concerns but also contribute to economic and social sustainability, making them a cornerstone of future green technologies. Grass fibers are mostly cheap and widely abundant natural fibers across the continent, research and development on these fibers and their related composites is currently at an all-time high.^8–10^

**Fig. 1:**
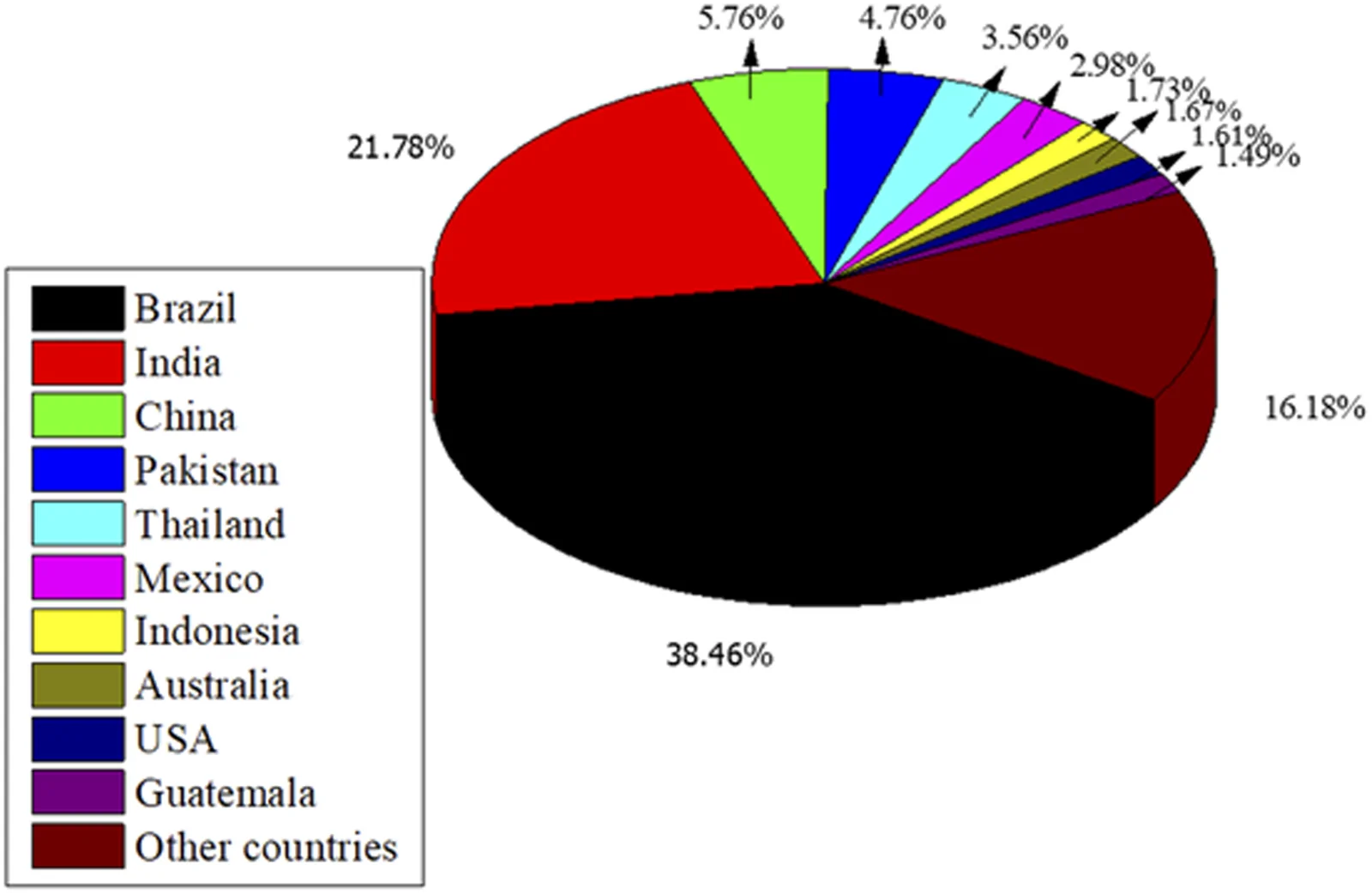
Annual global production of sugar cane from top ten countries.^13^

The sugarcane (*Saccharum officinarum*) plant, a species of tall, perennial grass, is the source of bagasse, or sugarcane grass fiber, which is made up of 32–45% cellulose, 20–32% hemicellulose, and 17–32% lignin.^11^ In addition to being a commercial crop, sugarcane leaf and wastes are fed to animals. Bagasse, a fibrous residue, is left behind after the harvested stalks' juice is extracted for sugar. Indigenous to the tropics and sub-tropics, sugarcane is the most productive crop in the world, with Brazil producing 38.46% of the world's total. Sugarcane contributes for 79% of global sugar production followed by sweet beets.^12^ With notable contributions from places like China and India, its use grew in popularity as sugarcane production extended throughout the world. However, Brazil is the world's top producer of bagasse followed by India with 21.78% and China with 5.76%.^13^ A bar diagram representing top ten countries of sugarcane production and their percentage is shown in Fig. 1.

**Fig. 2:**
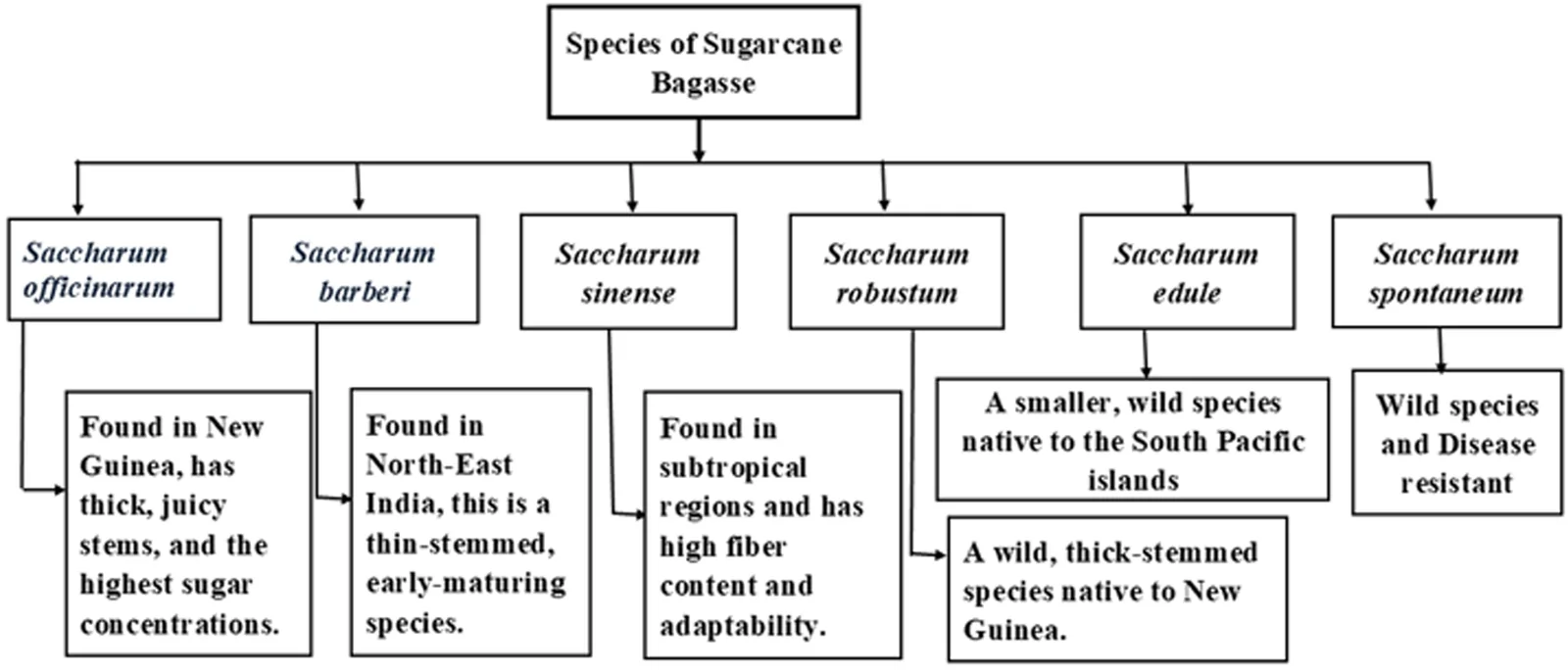
Different species of sugarcane available in the world.^14^

There are six identified species (Fig. 2) of sugarcane plant in the genus *Saccharum*, and each species has unique traits.^14^

**Fig. 3:**
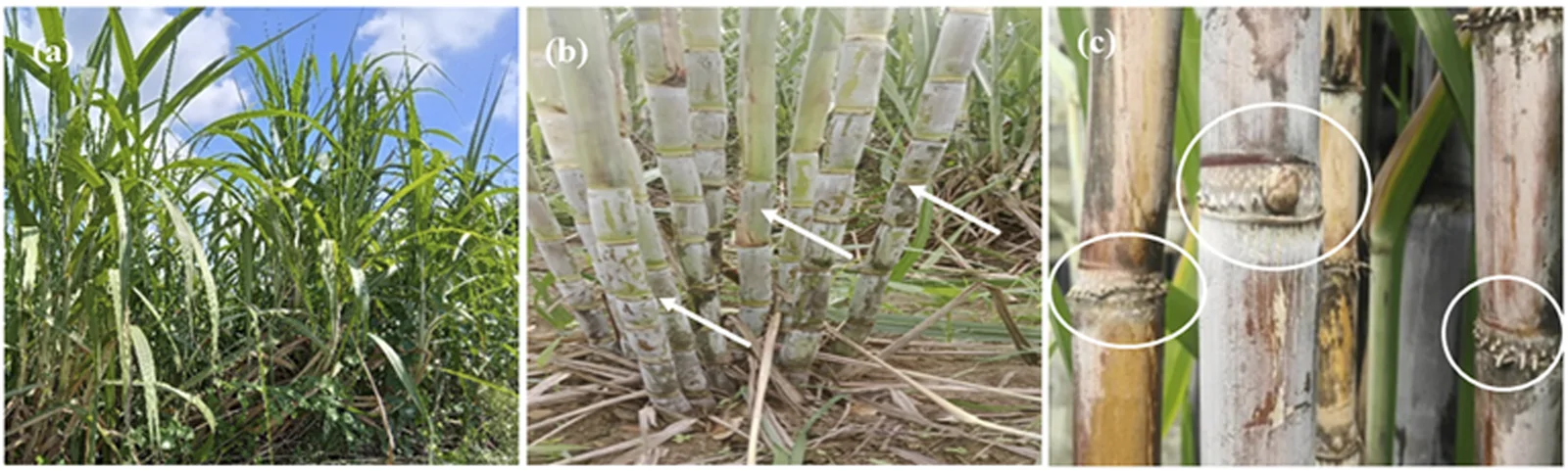
(a) Sugarcane plant (leaves), (b) stems (marked), and (c) nodes (marked).

After harvest, sugarcane components are frequently utilized as organic fertilizer and mulch in fields or as animal feed. Additionally, dried leaves can be used as thatch or burned for production of bioenergy. In order to anchor the plant and absorb moisture, sugarcane has a highly developed fibrous root system that extends deeply into the earth and consists of both shoot and sett roots.^15–17^ Digital photographs of sugarcane plant, nodes, and leaves are given in Fig. 3.

### 1.1. Extraction of bagasse fiber from sugarcane stem

**Fig. 4:**
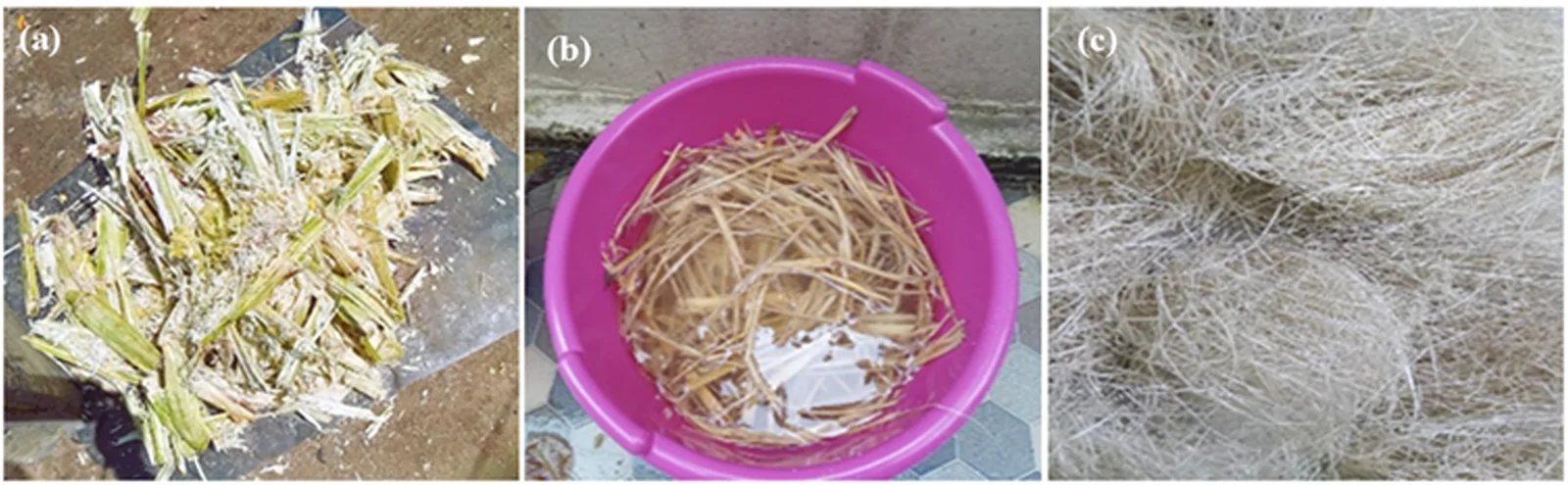
Digital photographs of (a) sugarcane residue, (b) retting of sugarcane, and (c) extraction of bagasse fiber from sugarcane.

The fibrous residue that remains after sugarcane juice is extracted is used to make bagasse fiber, a natural and environmentally favourable substance. The main techniques used to accomplish this are mechanical, chemical, retting, and advanced (by bleaching or steam explosion). The mechanical approach entails beating the bagasse with depithing machines (using dry, wet, or semi-dry methods) to separate the longer, durable bast fibers from the brittle pith, which is spongy, non-fibrous plant matter.^18,19^ The best-quality fibers are then isolated for additional processing by combing and sieving the separated raw material. A chemical procedure is necessary to dissolve the binding agents, namely lignin and hemicellulose, in order to obtain pure, individual cellulose fibers (nanocellulose). Bagasse fibers are boiled in a mild alkaline (sodium hydroxide) solution at temperatures ranging from 60 to 100 °C for 1 to 4 hours.^20^ This process eliminates lignin and hemicellulose, leaving high-quality raw cellulosic fiber. The retting technique is putting bagasse in water and letting it soak for several days to allow spontaneous bacterial decomposition to take place. While this method takes longer and results in slightly coarser fibers than chemical processes, it is significantly less expensive and eliminates chemical waste.^21^Fig. 4 depicts a schematic visual presentation of bagasse fiber extraction from discarded sugarcane using the retting method. Advanced approaches involve exploding the bagasse in an autoclave with high-pressure saturated steam (*e.g.*, 13 bar, 195 °C) for ten to fifteen minutes, followed by an abrupt pressure decrease. In order to effectively separate the fibers, this explosive decompression breaks up the plant structure.^22^ Enzyme pre-treatment procedure is also an environmentally acceptable methods to add enzymes (like xylanase) to pre-treat the bagasse before bleaching. Although different studies used different methods for fiber extraction, the fundamental processes were mostly the same. Every technique has pros and cons. For instance, the physical and autoclave methods use less energy but require less chemicals, the retting method takes longer, and the enzyme process uses more chemicals to extract bagasse fiber.^23,24^

In order to recover the bagasse fibers from the sugarcane rind, Collier *et al.*, (1992) first separated the rind from the pith and then removed the nodal regions, which have distinct physical characteristics. For one to two hours, they prepared the fibers with sodium hydroxide (NaOH) at varying concentrations (0.1 N and 1 N) at high atmospheric pressure (2 atm.).^25^ Bagasse fiber was pretreated in distilled water and salt at three different NaOH levels (2 N, 1 N, and 0.1 N) using the combing and retting methods, respectively. Following the extraction of the fibers using these techniques, it seemed that the chemical method had a low yield of around 40% while the natural methods (retting and combing) had a greater yield of about 70%.^26^ Using low-frequency ultrasonication to recover cellulose nanofiber (CNF) from bagasse, Shahi *et al.*, (2020) first treated hydrogen peroxide, which converted brown bagasse into cellulose-rich creamy white pulp. Alkaline circumstances disrupted the intermolecular ester link between lignin and carbohydrate. The second step is to ultrasonically process the cellulose. This step was taken to optimize the ultrasonication time, which ranged from 1 to 3 hours, as well as the influence it had on the fiber composition and structure.^27^

In order to treat bagasse at 130 °C for an hour in an autoclave, two strengths of NaOH (0.1 N and 1 N) was utilized. Two samples of bagasse were pretreated in an autoclave at the same temperature using distilled water and salty water, respectively. Pretreatment with simply distilled water (with 0.1 N NaOH) produced fibers with the maximum average length of 45.6 mm but higher linear density, whereas treatment with salty water (with 1 N NaOH) produced fibers with the lowest average length of 29.8 mm but finer fiber bundle.^28^ Following similar procedure, Chiparus and Chen (2003) extracted bagasse fiber of lengths ranging from 50.2 mm to 70.2 mm.^29^ In another study, bagasse fiber was soaked in 1% NaOH solution at 25 °C for 2 hours, keeping the liquor and fiber ratio at 20 : 1. The NaOH treatment increased tensile strength of fiber by approximately 17% and fiber length was found 0.9 cm.^30^ The mild alkali treatment found suitable chemical process to extract fiber from sugarcane waste.

### 1.2. Composition and properties of bagasse fiber

The exact breakdown of bagasse fiber varies slightly depending on the cane's maturity, soil type, and the efficiency of the sugar mill's crushing process. Bagasse is the fibrous byproduct remaining after the extraction of juice from sugarcane. Bagasse fiber consists mainly of cellulose (40–50%), hemicellulose (20–30%), and lignin (20–25%).^31^ Cellulose is the dominant component responsible for the mechanical strength of bagasse fiber due to its crystalline structure and high tensile capacity, while hemicellulose and lignin act as secondary components that influence flexibility, toughness, and durability.

**Fig. 5:**
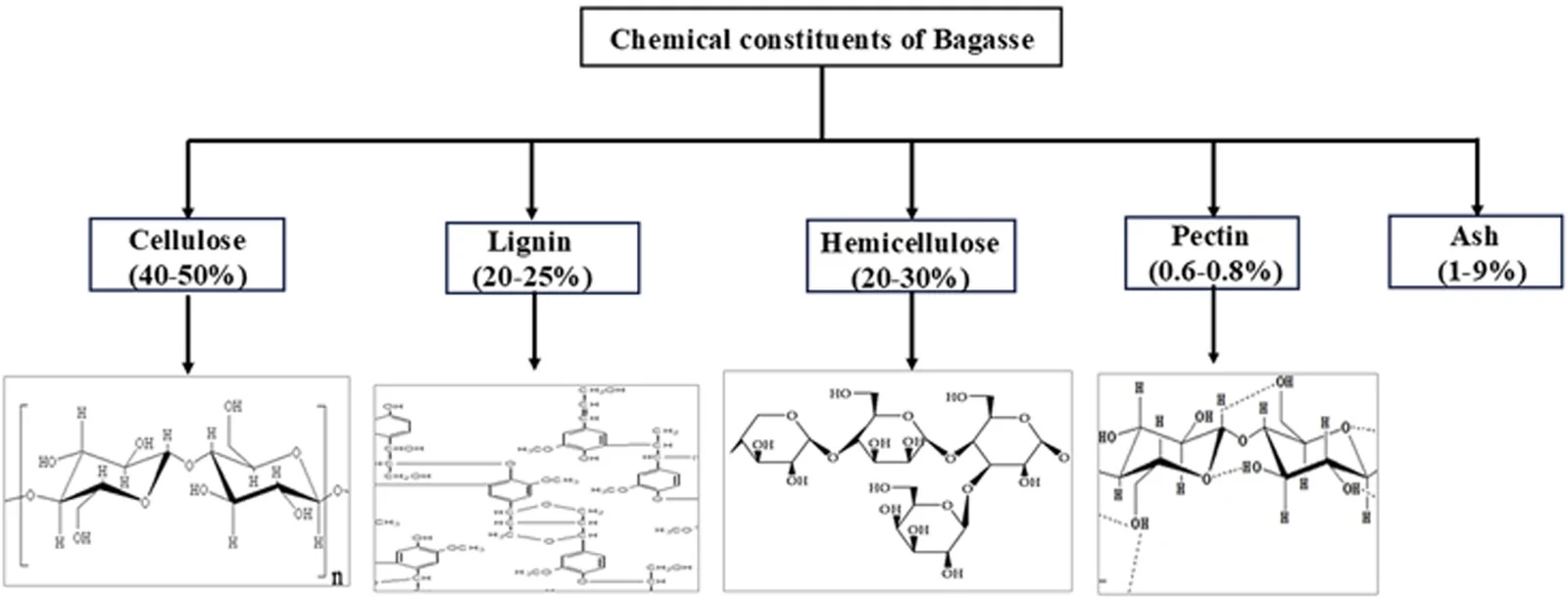
Composition of bagasse fiber.

The detailed composition of bagasse fiber is shown in Fig. 5 and different physical and mechanical properties of fibers are reported in Table 1, respectively. Bagasse possesses significant tensile strength (up to 300 MPa) and provides excellent structural reinforcement when blended with polymers. It also shows young's modulus of roughly 2.5 to 18 GPa depending on fiber treatment.^32^ It adds notable stiffness and impact resistance when used as reinforcement in composite materials due to presence of lignin and cellulose.

**Table 1:** Physical and mechanical properties of bagasse fiber after extraction

Method of extraction	Tensile strength (MPa)	Tensile modulus (GPa)	Elongation at break (%)	Water sorption after 24 hours (%)	Reference
Milling	290	17.0	—	—	34
Grinding	350	62.0	7.9	35	35
Retting	290	27.1	1.1		36
Retting	7.3	—	—	—	37
Coagulants	427	13.0	15	—	38
Retting	350	6.0	—	—	39

Bagasse fiber has a low bulk density (typically 0.40 to 0.55 g cm^−3^). The fibers have a corrugated surface and irregular lumens, which give them excellent oil-absorbing and fluid-retention capabilities. Its high cellulose content gives it a natural crystalline structure, while lignin acts as a natural binder and adhesive. The fiber also contains small amounts of wax and ash.^33^

### 1.3. Surface modification of bagasse fiber

The main goals of surface modification for bagasse fiber are to roughen the surface, eliminate surface waxes, and lessen the fiber's inherent hydrophilicity. This increases the strength and endurance of the resultant bio-composites by strengthening mechanical interlocking and interfacial adhesion with hydrophobic polymer. Chemical therapy (alkali and silane treatment, acetylation, esterification, *etc.*), biological treatment, physical treatment, *etc.* are common and very successful surface modification methods are reported recently.^40–42^ Surface treatments remove impurities, waxes, and hemicellulose, thereby exposing additional hydroxyl groups. This enhances fiber–matrix adhesion and promotes more efficient stress transfer under gradual tensile loading, enabling the composite to sustain higher tensile stresses before failure.

Bartos *et al.*, (2021) analyse the effects of the alkali treatment (NaOH) on the composition, structure, and properties of the bagasse fibers and reported that the cellulose content peaked at an intermediate concentration, while the hemicellulose and lignin contents decreased as the alkali concentration rose.^40^ The chemical treatment of bagasse at 6%, 12%, and 18% NaOH concentrations were investigated by Ait-abdellah *et al.*, (2024) and found at 18%, fiber degradation occurred due to removal of hemicellulose, lignin that was validated by FTIR and solid-state ^13^C NMR examinations. With 6% alkali treatment the fiber shows optimum balance between surface modification and mechanical integrity, leading its strength and surface quality.^41^ Alkali, oxalic acid, and silane were applied to bagasse fiber separately in one of research work, which showed that alkali treatment increased the fiber density from 1.266 to 1.311 g cm^−3^ whereas silane treatment only improved to 1.281 g cm^−3^, and oxalic acid to 1.272 g cm^−3^ respectively. The crystallinity indices were found 43.94% (silane-treated), 44.08% (oxalic acid), 47.85% (alkali) compared to that of 41.21% for untreated fibers.^42^ The impact of 2,2,6,6-tetramethylpiperidin-1-oxyl (TEMPO) and NaOH on mechanical properties, thermal stability, and crystallinity of bagasse fiber was investigated and reported that the crystallinity rose from 50.95% (raw fiber) to 58.38% (NaOH treated fiber) and 52.34% (TEMPO treated fiber). In mechanical tests, TEMPO-treated fibers gave the highest tensile strength of 141.10 MPa and Young's modulus of 4495.86 MPa respectively where NaOH-treated fibers showed a lower total degradation rate (75.6%) compared to former (77.7%) in thermogravimetric analysis.^43^

**Fig. 6:**
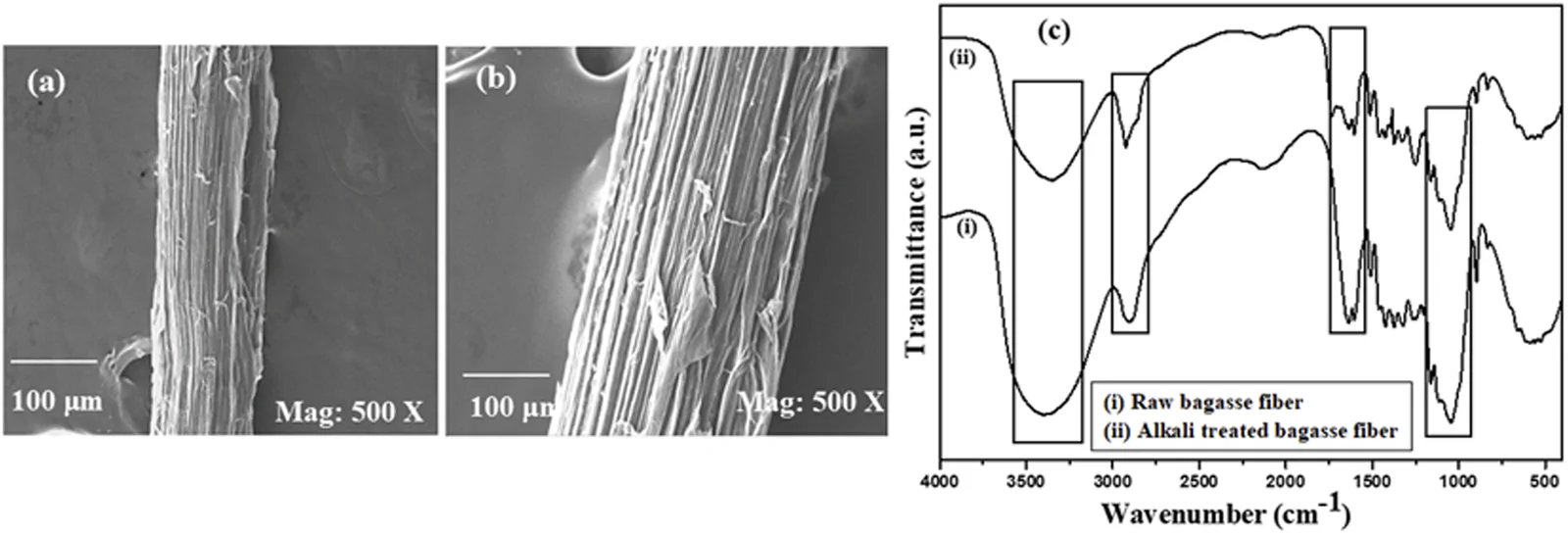
FE-SEM micrographs of (a) bagasse fiber, (b) alkali treated bagasse fiber, and (c) FTIR graph of raw, and alkali treated bagasse fiber.

Using NaOH solutions (1% to 4%) and different immersion times (2 to 24 hours), Hajizadeh *et al.*, (2025) examined the effects of alkaline treatment and stated 3% NaOH solution with an 8 hours immersion, produced an increase in the sound absorption coefficient property of fiber whereas above 4% distorted the fiber shape which reduced its performance and structural integrity.^44^ The similar experiment was carried out by another researcher, who found soaking time and NaOH concentration affect bagasse fiber tensile strength. FTIR spectroscopy confirmed the partial removal of lignin and hemicellulose at higher alkali treatment.^45^ Bagasse fiber was treated with a low alkali concentration (2 wt%) for two hours and found an increase in surface area (Fig. 6(b)) compared to that of raw bagasse fiber (Fig. 6(a)) by FE-SEM micrographs and a shift in wavenumbers in the FTIR graph (Fig. 6(c)), respectively. A distinctive broad peak for –OH stretching was seen in raw bagasse between 3200 and 3500 cm^−1^, and its strength dropped following alkali treatment and the peak's intensity decreased because of breaking of the hydrogen bonds between cellulose and hemicellulose. Alkali treatment of fibers results in reduced intensities of the absorption bands near 1640 cm^−1^ and 2920 cm^−1^, corresponding to the acetyl and uronic ester groups of hemicellulose, the ester linkages of lignin, and the aliphatic C–H stretching vibrations of cellulose and hemicellulose. This spectral change indicates partial removal of hemicellulose and lignin components. The absorption band observed around 1030–1100 cm^−1^, characteristic of the polysaccharide backbone and cellulose structure in raw fibers, appears more pronounced in alkali-treated fibers. This enhancement reflects the relative enrichment of pure cellulose as non-cellulosic constituents are progressively removed during treatment.^46^

Cellulose was recovered from sugarcane bagasse both with and without acid hydrolysis using soda-anthraquinone, and hydrogen peroxide or salt chloride. For bagasse without hydrolysis, the pulping output was 34.6%, and with hydrolysis, it was 26.6%.^47^ Wang *et al.*, (2020) treated bagasse with hydrogen peroxide (H_2_O_2_) and phosphoric acid (H_3_PO_4_) to develop CNF. Bagasse fiber yields of 93% and 50% of CNF, respectively due to above chemical treatments.^48^ The impact of potassium dichromate (K_2_Cr_2_O_7_) and potassium permanganate (KMnO_4_) modification on bagasse fibers was reported which informed that crystallinity index was dropped from 72% (raw fiber) to 50% (K_2_Cr_2_O_7_) and 47% (KMnO_4_) due to elimination of polar impurities.^49^ The effects of various concentration of H_2_O_2_ (0, 10, 20% w/w) and NaOH (10, 20, 40% w/w) on the surface of bagasse fibers were treated and summarized that with the highest alkali charge without peroxide produced the highest hemicellulose precipitation (yield of 85%). Alkali charge considerably raised the hemicellulose extraction yield in an alkaline peroxide treatment of bagasse with metal ion control and an inert atmosphere.^50^ Chemical treatments are effective but environmentally taxing due to effluent generation and toxicity, whereas physical and biological methods are cleaner, safer, and more sustainable. Biological (enzymatic) approaches, in particular, align with green chemistry principles, though they require further optimization for industrial scalability.

Without the use of harsh chemicals, physical treatment like heat treatment, ultrasonication, and steam explosion are important to improve bonding of treated fiber when employed as reinforcement in polymer composites. In a steam explosion, the biomass is exposed to high-pressure steam for a brief period of time. The lignocellulosic matrix is broken by this thermo-mechanical shock, which separates cellulose fibers and alters lignin.^51^ To improve the surface area available for enzymatic degradation, the fiber is mechanically ground into smaller particles. The ultrasonication process requires high-frequency acoustic waves to travel through a water medium containing bagasse. This cavitation process cleans the fiber surfaces, reduces aggregation, and improves surface roughness, resulting in improved fiber–matrix interaction.^52^ Heating fibers at 120 to 250 °C for 10 minutes to several hours can changes the cell wall structure and partially degrades hemicellulose, which reduces the fiber's water soaking property and makes it more suitable for interaction with hydrophobic thermoplastic matrices. In radiation pretreatment, fibers are exposed to gamma rays or electron beams, that breaks down cellulose crystallinity and relaxes the structural network of the biomass.^53,54^Table 2 reports the changes in fiber properties after various surface treatments on bagasse fiber.

**Table 2:** Different properties of bagasse fiber after various surface treatment^a^

Surface treatment condition	Mechanical properties (MPa)	Water sensitivity	Thermal property (°C)	Other property	Reference
Raw fiber	TS: 20–50, TM: 2700	WA:17.8%	TMS: 200	BD:1.88 g cm^−3^	55
Silane (temp.: 200 °C; coating time: 2 h)	—	WA:10%	—	BD: 3.48 g cm^−3^	56
Acylation (temp.: 80 °C; coating time: 48 h)	—	—	TMS: 322	CI:37.1%	57
10% NaOH + 5% KMnO_4_ (R.T.; time: 0.5 h; pH:7)	—	—	TMS: 300	CI: 55.2%	58
Steam explosion (steam temp.: 203 °C for 3 min.)	—	—	—	BD: 0.695 g cm^−3^	59
Oxygen plasma treatment + 20 wt% cellulose/TS-POSS (voltage: 0.1 Kw; etching time: 500 s; pressure: 25 Pa.)	—	CA: 152.9°	TMS: 364	—	60
1% Acrylic acid (R.T. for 1 h.)	TS:229.01, TM: 8090	—	—	BD: 1.59 g cm^−3^	61

a TS-POSS: triSilanolIsobutyl-polyhedral oligomeric silsesquioxane; TS: tensile strength; TM: tensile modulus; WA: water absorption; CA: contact angle; TMS: thermal stability; BD: bulk density; CI: crystallinity index.

### 1.4. Development in bagasse fiber reinforced thermoplastic composites

A thorough examination of bagasse fiber reinforced thermoplastic matrix-based composites, shows increasing interest by the researcher due to former's mechanical properties and cost benefits. Different thermoplastics such as polyethylene, polypropylene, and phenol, along with thermosetting *i.e.*, epoxy and polyester have been extensively studied as the matrix material, along with bagasse fiber to develop biocomposites.^62–66^ The size of the reinforcing fiber, which can be as small as micro- or nano-scale particles, has a major impact on the mechanical, chemical, thermal, and moisture absorption properties of these bagasse composites. Studies show that improving fiber surface treatment techniques, fiber content, and the way the composites are made can greatly improve their performance, making bagasse fiber-reinforced biocomposites a practical substitute for traditional synthetic materials.^67,68^

Youssef *et al.*, (2008) combined bagasse fibers with various thermoplastics such as low-density polyethylene (LDPE), high-density polyethylene (HDPE), polystyrene (PS), polypropylene (PP), and polyvinyl chloride (PVC). Their findings indicated that LDPE and HDPE were more effective in reducing the water absorption of the composites compared to that of the PS, PP, and PVC. On the other hand, treatments like electron beam (EB) or poly(ethylene terephthalate) (PETA) had minimal impact on HDPE and PVC.^62^ Bagasse cellulose (10 wt%) reinforced HDPE composites were developed which showed lower tensile strength than pure HDPE due to poor adhesion between the fibers and the matrix might be due to cellulose agglomeration. Modifying the cellulose decreased elongation by 26% but increased tensile modulus by 50%.^63^ To study the impact of EB irradiated fiber content in LDPE and HDPE composites, the researcher observed mechanical and physical strength declined only when the fiber content surpassed 50 wt% whereas water absorption was not significantly affected.^64^ Chen *et al.*, (2021) utilized recycled HDPE as the matrix and bagasse fibers pretreated by washing, mercerization, and acetylation to enhance adhesion, which led to improvements in tensile and flexural modulus.^65^ Using recycled polyethylene as resin uncarbonized and carbonized bagasse particles (10–50 wt%) reinforced biocomposites were developed, which achieved the best mechanical properties at 30 wt% loading of carbonized bagasse.^66^ Taking recycled HDPE, bagasse composites were developed through melt mixing and compression moulding, scanning electron microscopy (SEM) images showed good fiber–matrix contact at the interface of the composite.^67^

Jimenez *et al.*, (2017) reinforced bagasse using PP, HDPE, and starch-based matrices, achieving an optimized Young's modulus of 3.413 GPa, which was validated through micromechanical models.^68^ Bagasse fiber reinforced epoxy composites were developed which showed highest tensile strength value of 17.98 MPa only, due to the creation of voids and fiber agglomeration within the matrix.^69^ Hybrid epoxy composites reinforced by bagasse and coir fibers showed property differences based on fiber ratios compared to pure bagasse or coir composites.^70^ Kennedy *et al.*, (2025) tested epoxy and polyester composites reinforced with 30% alkali-treated bagasse fibers for terrace coatings. Epoxy composites had better tensile strength of 39.46 MPa and flexural strength of 65.92 MPa because of improved fiber–matrix bonding, while polyester composites showed better thermal stability, making them suitable for applications sensitive to temperature.^71^Table 3 represents the mechanical properties of some bagasse and thermoplastic matrix-based composites which developed recently.

**Table 3:** Properties of bagasse fiber reinforced thermoplastic resin-based composites^a^

Matrix	Techniques	Tensile strength (MPa)	Tensile modulus (MPa)	Flexural strength (MPa)	Flexural modulus (MPa)	Reference
PP	Compression	17	1600	—	—	72
PP	Injection	23.0	1027.1	35.5	960.7	73
PVC	Compression	52	—	—	—	74
Polyethylene	Injection	20.1	489	26.2	996	75
Epoxy	Hand lay up	9.9	—	22.7	—	76
PP	Mixer	19.5	980	—	—	77
Polyester	Compression	12.5	1534	—	—	78
Polyester	Hand lay up	77.8	10.32	210.4	21.10	79
Epoxy	Hand lay-up	25.5	—	—	—	80
Polyester	Compression	12.7	—	—	—	81

a PP: poly(propylene); PVC: poly(vinyl chloride).

Anggono *et al.*, (2017) investigated sugarcane fibers as reinforcement in polypropylene-based biocomposites. Tensile test showed that using 1 : 3 weight ratio of fiber to matrix raised tensile strength of composite to 24.92 MPa. In contrast, Ca(OH)_2_ treatment on fiber surface reduced the tensile strength to 11.30 MPa.^82^ Bagasse fiber was employed in PVC resin to enhance waste management strategies. A maximum tensile strength of composite of 25–27 MPa and modulus around 240 MPa were achieved. Biodegradation analysis showed that after five weeks under soil burial, the composite they lost only 13% of its original mass.^83^ With thermoset phenolic resin, sugarcane bagasse fibers, demonstrated potential composite for non-structural applications *i.e.*, packaging and vehicle interiors.^84^

**Fig. 7:**
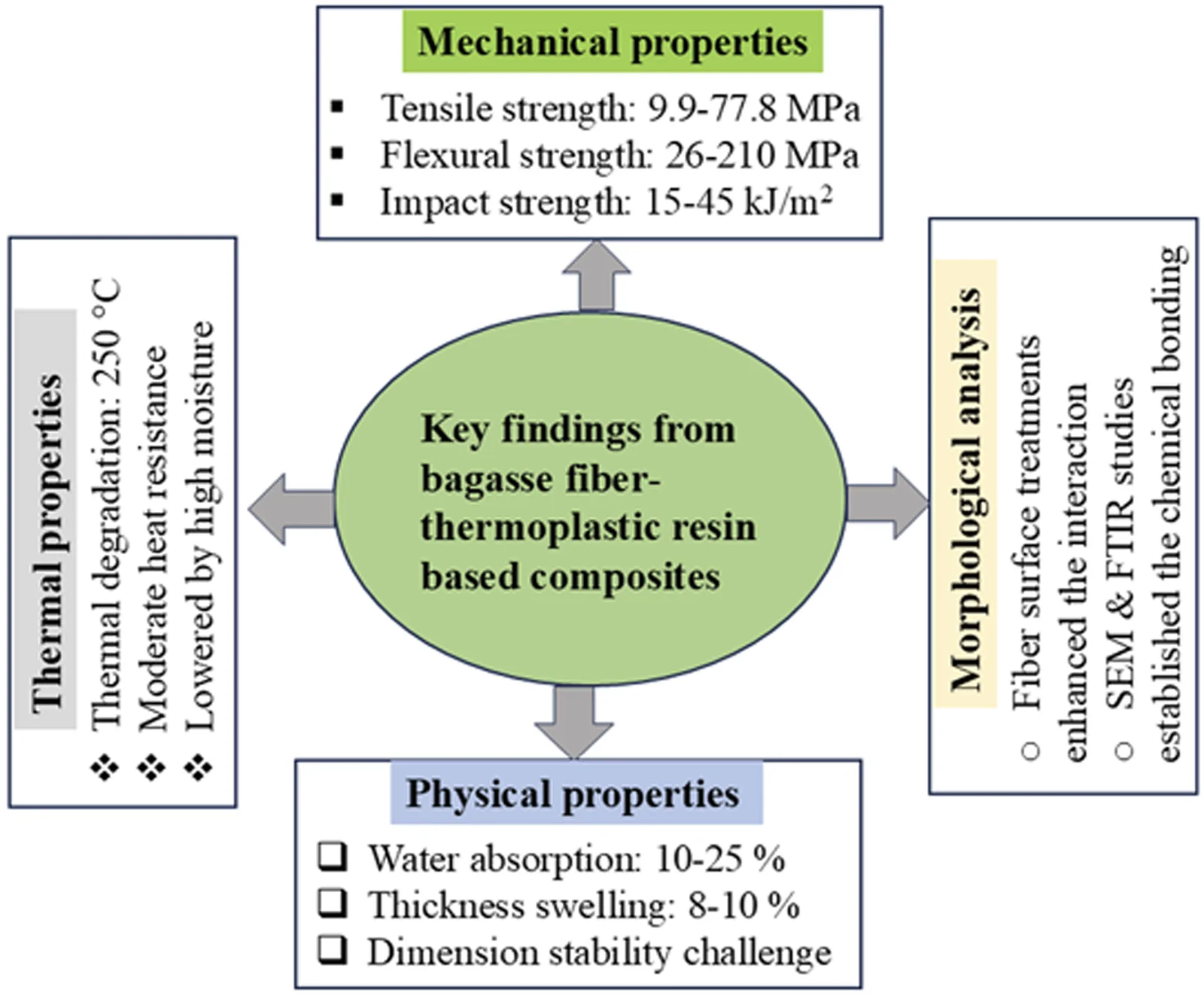
Overview of literature studies on bagasse fiber-reinforced composites, highlighting the key findings of their physico-mechanical properties.

Overall, bagasse fiber–reinforced thermoplastic resin-based composites exhibited promising physico-mechanical properties, including moderate tensile strength (4–85 MPa), flexural strength (26.2–210 MPa), and thermal stability up to 200–250 °C. Hydrophilic bagasse fibers are chemically incompatible with hydrophobic polymers because their polar, moisture-absorbing surfaces cannot bond effectively with non-polar matrices. This polarity mismatch and moisture sensitivity result in weak interfacial adhesion and poor composite performance unless surface treatments or compatibilizers are applied. However, challenges like high moisture absorption and weak interfacial bonding still exist. Many studies show that chemical and surface treatments greatly enhance these properties, making bagasse composites appropriate for use in automotive, packaging, and construction sectors. Fig. 7 provides a summary of the recent research on the properties of bagasse fiber-reinforced biocomposites.

### 1.5. Bagasse fiber reinforced bioresin based composites

**Fig. 8:**
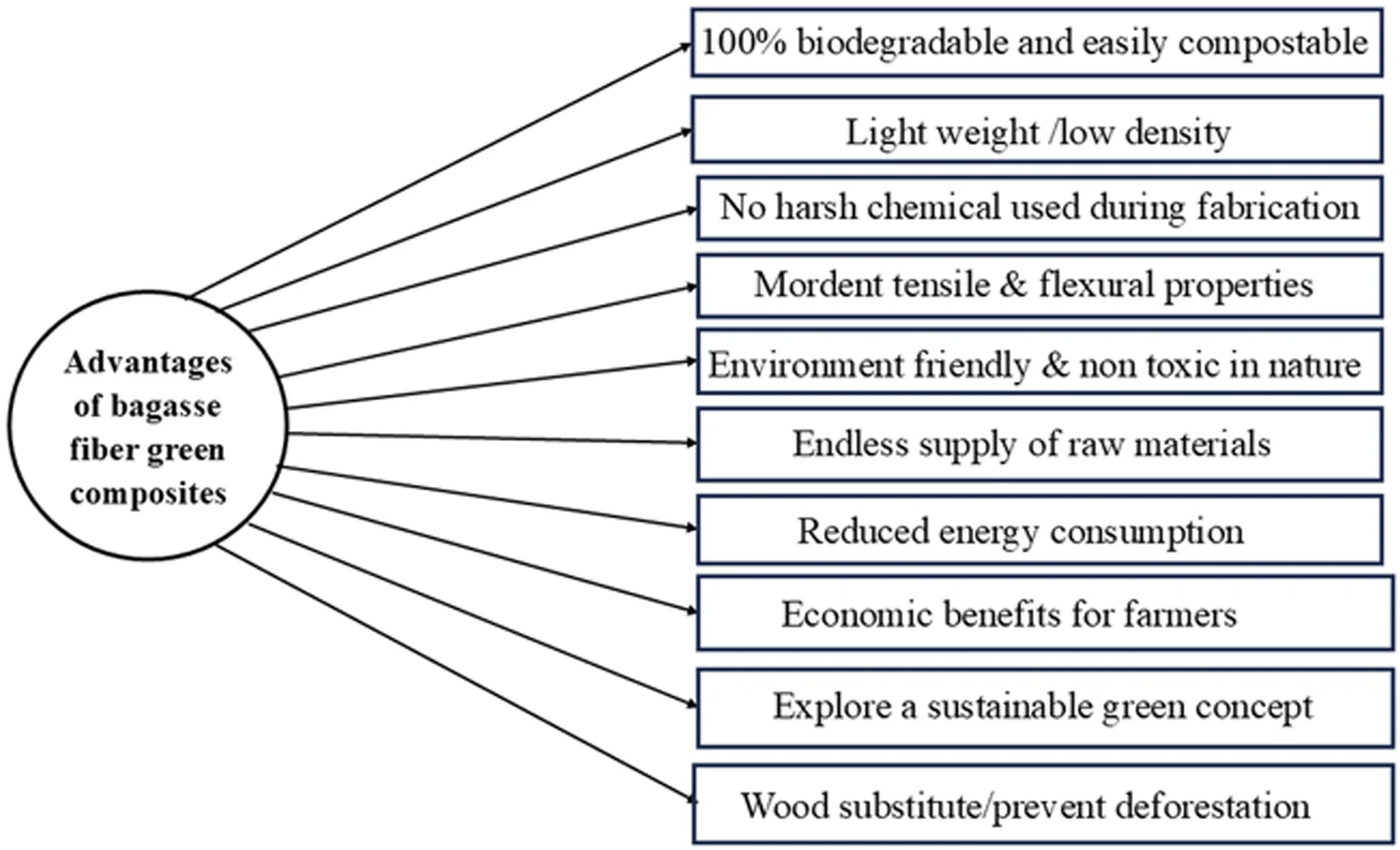
Advantages of sugarcane fiber reinforced completely biodegradable composite.

Bagasse fiber-reinforced bioresin composites are prepared by combining agricultural waste from sugarcane stalks with plant-based bioresins such as starch, bio-epoxy, and polylactic acid (PLA).^85–88^ These materials are environmentally friendly and have a good strength-to-weight ratio, improved resistance to wear, and effective thermal insulation properties. They serve as sustainable substitutes for synthetic plastics in areas like packaging and automotive panels, as illustrated in Fig. 8.

Asrofi *et al.*, (2020) developed tapioca starch-based sugarcane bagasse composite, which achieved improved moisture resistance (28.3% at 22.5 hours) and tensile strength (20.84 MPa).^85^ Similarly, a biocomposite made up of bio-polyethylene and bagasse pulp showed enhanced mechanical properties at 40 wt% fiber loading and reduced CO_2_-equivalent emissions through bio-based compatibilizers.^86^

**Fig. 9:**
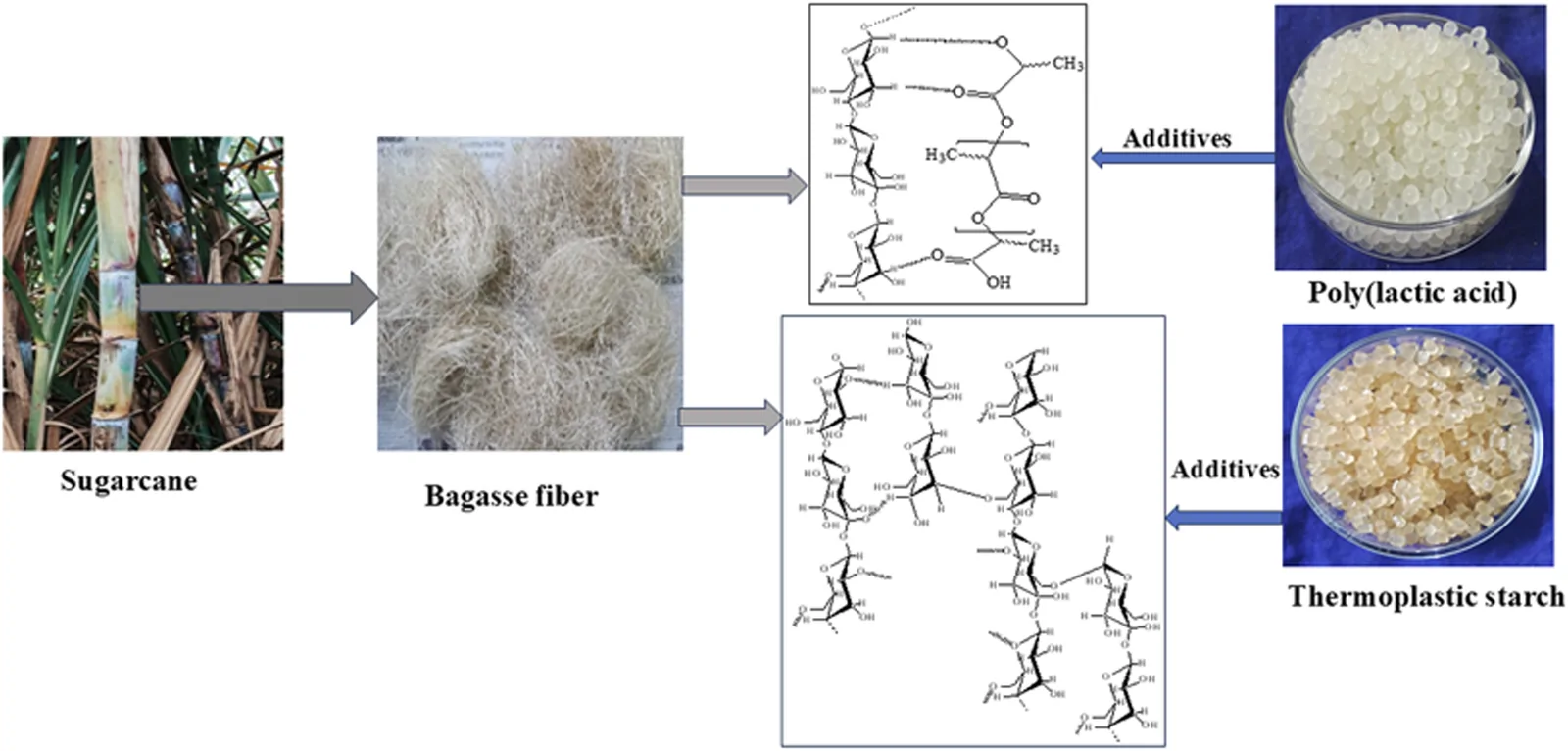
Schematic interaction between cellulosic hydroxyl group with functional group of bioresin.

Balaji *et al.*, (2017) fabricated completely biodegradable composites with sugarcane bagasse fibers and cardanol, a bio-based substance obtained from cashew nut shell liquid. The composite reinforced with 15 wt% fiber exhibited the best tensile performance, along with good fiber dispersion, incorporation, and adhesion within the cardanol epoxy matrix.^87^ Mattos *et al.*, (2024) proposed an alternative approach for synthesizing and utilizing bio-oil-based benzoxazine resins in the production of “green” medium-density fiber board. Their work demonstrated improved mechanical properties, with the modulus of rupture reaching 15 MPa.^88^ Using four polymer matrices *i.e.*, PLA, HDPE, bio-epoxy (BE), and unsaturated polyester resin (UPR), Phiri *et al.*, (2025) developed sugarcane bagasse composite which showed that matrix type and fiber loading have a significant impact on mechanical behavior of composite. 6 wt% BE have the highest tensile modulus and impact strength, while 3 wt% PLA achieved peak flexural strength, and 9 wt% PLA had the highest flexural modulus.^89^ The use of bioresin improved the flexural behaviour of bagasse composite due to the strong interaction between the functional groups of bioresin with functional group of cellulosic bagasse fiber (as shown in Fig. 9) to develop a strong and rigid composite.

Asrof *et al.*, (2025) fabricated PLA-based biocomposites reinforced with bagasse fibers using 3D printing technology. The study examined the effect of alkali treatment which informed 6% NaOH-treated fibers achieved the highest tensile strength of 34.59 MPa, flexural strength of 45.62 MPa, and impact strength of 45.03 kJ m^−2^, respectively. These results emphasize the importance of selecting the right matrix and treating fibers effectively to achieve the best mechanical properties. The study provides valuable insights into designing sustainable materials from agricultural waste, particularly for use in packaging, construction, and automotive industries.^90^ Few researchers have reported regarding the use of bioresin with bagasse fiber to develop completely biodegradable composites. Some physico-mechanical and thermal properties of these composites are reported in Table 4. Because of their improved performance and ability to break down naturally, PLA/bagasse fiber biocomposites have significant potential for use in everyday products, car components, and other environmentally friendly material applications.

**Table 4:** Properties of bagasse fiber reinforced bio resin-based composites^a^

Matrix	Thermal stability (°C)	Tensile strength (MPa)	Tensile modulus (MPa)	Flexural strength (MPa)	Flexural modulus (MPa)	Reference
Cardanol	480	36	—	74	—	91
Corn starch	—	35	1433.8	—	—	92
Soy based	—	9.22	759		1978	93
PLA	—	37.89	234.4	87.80	2795	94
Starch	—	4.15	162.50	—	—	95
Starch	245	85	—	—	—	96
P-Strach	250	4.44	16.17	—	—	97
Corn starch	—	—	—	44	2290	98
TPS	—	1.70	22.55	—	—	99
P-Strach	291	—	—	—	—	100
Bio-epoxy	320	71	3300	—	—	101

a PLA: poly(lactic acid); TPS: thermoplastic starch.

Sugarcane bagasse shows great potential in improving the mechanical, thermal, and eco-friendly qualities of composite materials. When treated chemically or combined with other reinforcing materials, bagasse fibers greatly enhance resistance to moisture, strength, and environmental benefits, making them a strong option for biodegradable plastics, lightweight biocomposites, and structural supports.^102^ However, there are still some challenges, including high water absorption, porous structure, and difficulty in scaling up production for industrial use. Future studies should concentrate on improving fire resistance, strengthening the bond between fibers and the matrix material, and ensuring long-term durability, which are essential for using bagasse-based composites in high-performance industries like automotive, construction, and packaging. By overcoming these issues, 100% biodegradable bagasse fiber composites (green composites) can move from being special eco-friendly materials to widely used sustainable alternatives, promoting innovation in circular economy strategies and decreasing dependence on synthetic plastics.^103,104^

### 1.6. Applications of bagasse fiber reinforced composites in different sectors

**Fig. 10:**
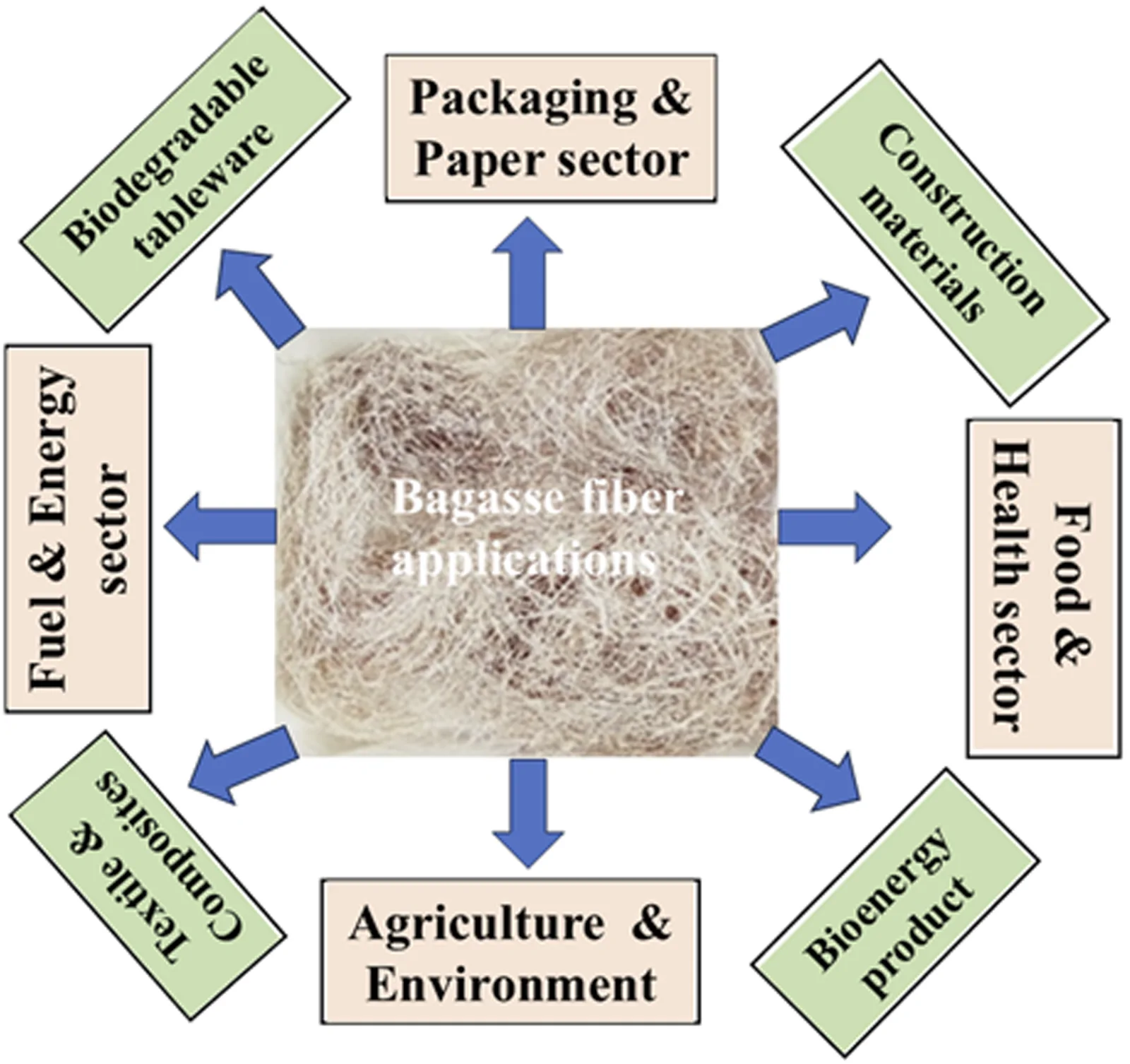
Application of bagasse fiber in different sectors.

Bagasse fibers are increasingly being used in cement, polymer, and hybrid composites as an environmentally friendly reinforcement. Its main uses are in the construction industry (particle boards and ceiling tiles), the automotive industry (lightweight interior panels and bumpers), and the production of ballistic armor. Bagasse fibers are mostly utilized in the following industries: (i) paper and packaging; (ii) biodegradable dinnerware; (iii) textiles and composites; and (iv) fuel and energy.^105,106^ Other sectors where bagasse fibers are utilizes is shown in Fig. 10.

**Fig. 11:**
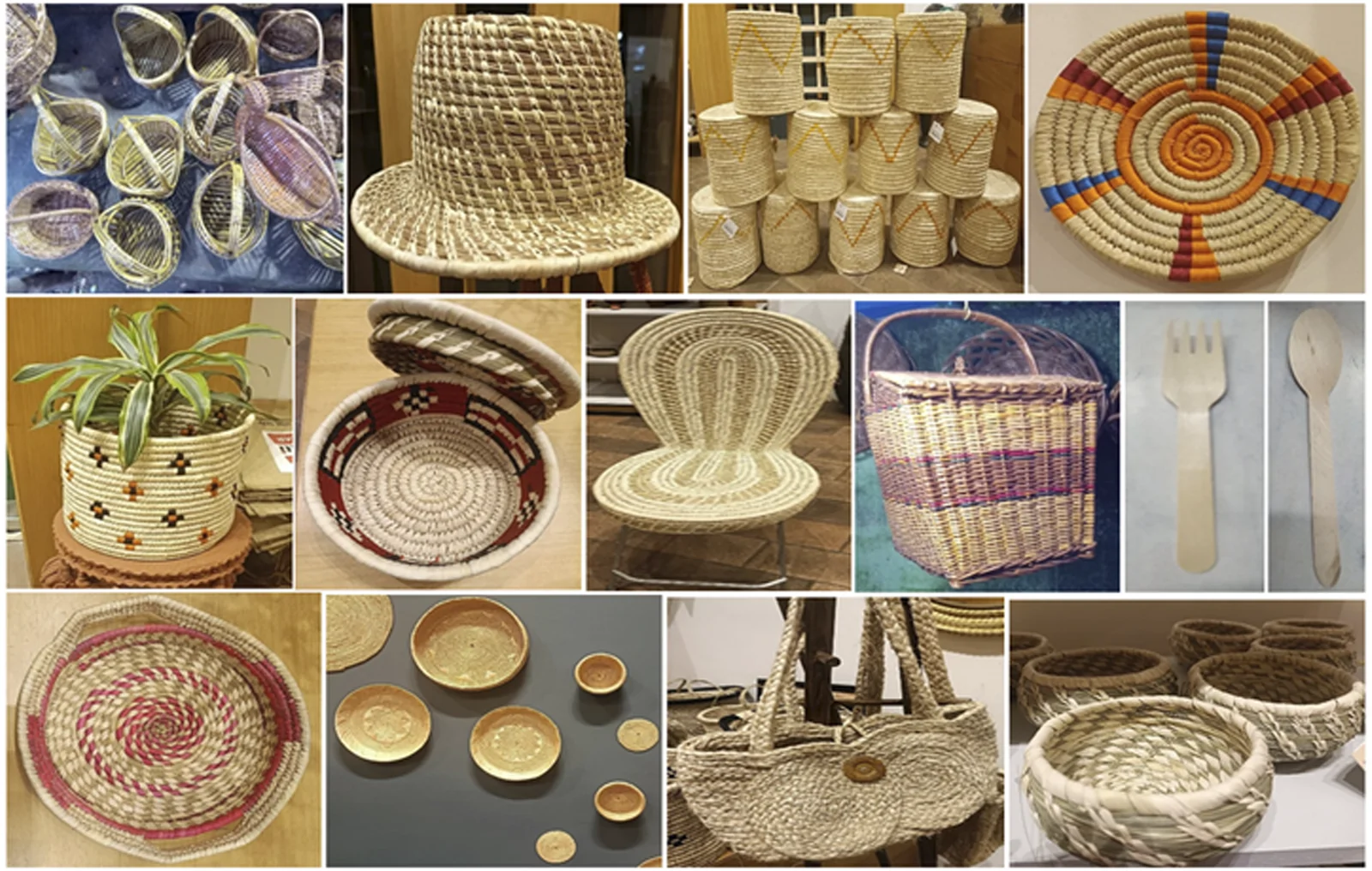
Various uses of bagasse fiber and its reinforced composites from household products to furniture.

Lila *et al.*, (2021) investigated the mechanical properties of bagasse fiber/polypropylene composites, emphasizing injection moulding circumstances and fiber mass fraction. Both injection and press moulding were used to create environmentally friendly composites reinforced with bagasse fiber, a natural byproduct of sugarcane processing. The matrices chosen were polypropylene and polycaprolactone–cornstarch, which stand for recyclable and biodegradable resins, respectively.^107^ Bagasse fiber-reinforced composites provide a compelling mixture of environmental friendliness, mechanical strength, and cost effectiveness, making them appealing for sustainable material development. There are several benefits of using bagasse fiber reinforced composites *i.e.*, environmental benefits, renewable and biodegradable, low carbon footprint, and waste valorization. Moderate mechanical & physical properties, low cost, and thermal insulation are other valuable advantages of bagasse fiber reinforced composites.^108,109^ The utilities developed from bagasse fiber and bagasse fiber reinforced composites available in the market are given in Fig. 11.

## 2 Future scope of the bagasse fiber and its reinforced composites

Many countries, including Brazil and India, are promoting ethanol-based petroleum (E-petrol) as a future fuel to reduce crude oil consumption. This shift will increase bagasse production, creating opportunities for its use in biocomposites.^110^ Future research should focus on developing effective, eco-friendly, and scalable pretreatment methods to enhance bagasse fiber–reinforced composites. The goal is to improve fiber compatibility with biodegradable polymer matrices while minimizing environmental impact.^111^ Promising green pretreatment technologies include enzymatic, microbial, deep eutectic solvents, ionic liquids, ozone, and plasma treatments, which can replace conventional chemical processes. The performance and sustainability of bagasse-based composites are anticipated to improve using these approaches.^112^

**Fig. 12:**
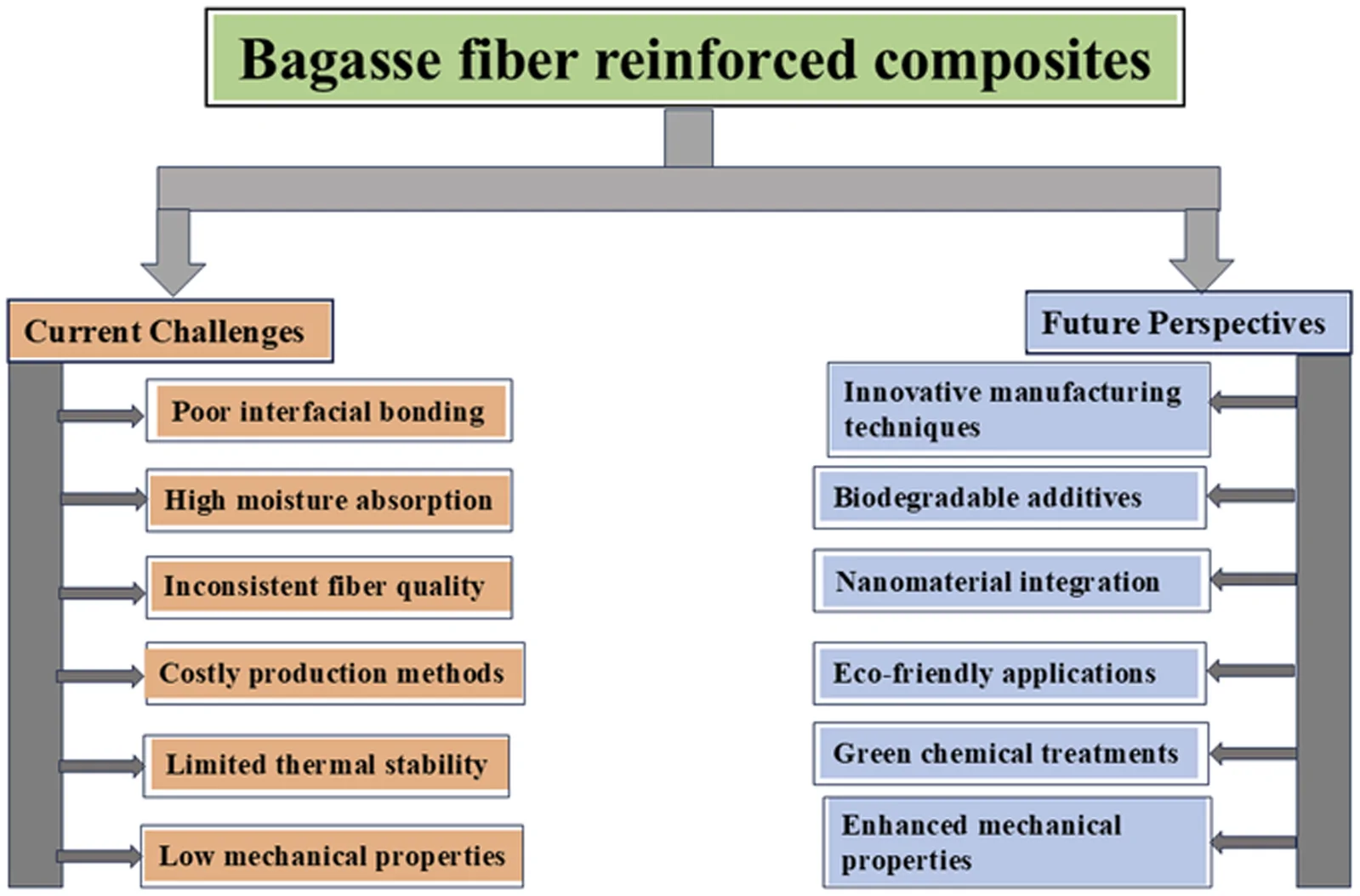
Current research trend and future directions for bagasse fibre reinforced composites.

Understanding structure–property correlations requires thorough research using contemporary analytical tools on fiber surface chemistry, crystallinity, and interfacial adhesion. Additionally, pretreatment parameters are optimized using statistical tools such as (i) response surface methodology and (ii) experiment design to optimize treatment settings such as chemical concentration, temperature, treatment time, pH, and fiber-to-solution ratio.^113^ Additionally, combined and sequential pretreatments that examine the synergistic effects of combined treatments, such as alkali addition with silane and steam explosion combined with enzymatic treatment, are investigated in order to improve fiber surface properties while reducing fiber damage.^114^ Additionally, more research should be done on the synergistic effects of various pretreatment techniques on mechanical, thermal, barrier, and biodegradation properties. Under order to ensure economic viability, future research should also concentrate on life cycle assessment, large-scale processing feasibility, and durability under realistic environmental conditions. High-performance, multipurpose, and completely sustainable biocomposites for packaging, automotive, construction, aerospace, and biomedical applications could be created by combining nanotechnology and bio-based functional additives with pretreatment bagasse fibers.^115,116^ Few current research challenge and future perspective for bagasse fiber reinforced composites is shown in Fig. 12.

## 3 Conclusions

Bagasse fiber reinforced polymer composites have become a potential sustainable material with advantages for the environment, cost effectiveness, and mechanical strength. The hand lay-up technique remains the most common fabrication method, though centrifugal and pultrusion moulding have also been explored. Inorganic fillers are commonly included in epoxy-based composites to improve their reinforcing properties, but issues related to cost and maintaining consistent quality continue to be a challenge. The chemical treatment of the surface of bagasse fibers has broadened their use in various industries such as automotive, packaging, furniture, and electronics. While compared with thermoplastic matrix-based bagasse composites which may exhibit higher mechanical strength than completely biodegradable bagasse composite, but formers incomplete degradability is a threat to environment. Green bagasse composites provide advantages such as design flexibility, recyclability, and lightweight performance. Among these, bagasse is especially notable due to its low cost, renewable source, and effectiveness in creating economical composite materials that can be widely utilized.

## Author contributions

Benudhar Parida: conceptualization, formal analysis, writing – original draft, writing-review & editing. Bijnyan Ranjan Das: supervision, conceptualization, methodology, review & editing. Ajaya K. Behera: supervision, visualization, formal analysis, writing – original draft, writing-review & editing.

## Conflicts of interest

The authors declare that they have no conflict of interest.

## Data Availability

No new data were created or analysed in this study. Data sharing is not applicable to this article.
